# Differential prognostic impact of CD8^+^ T cells based on human leucocyte antigen I and PD-L1 expression in microsatellite-unstable gastric cancer

**DOI:** 10.1038/s41416-020-0793-y

**Published:** 2020-03-17

**Authors:** Yoonjin Kwak, Jiwon Koh, Yujun Park, Yun Ji Hong, Kyoung Un Park, Hyung-Ho Kim, Do Joong Park, Sang-Hoon Ahn, Woo Ho Kim, Hye Seung Lee

**Affiliations:** 1 Department of Pathology, Seoul National University Hospital, Seoul National University College of Medicine, Seoul, South Korea; 2 Department of Pathology, Seoul National University Bundang Hospital, Seoul National University College of Medicine, Seongnam, South Korea; 3 Department of Laboratory Medicine, Seoul National University Bundang Hospital, Seoul National University College of Medicine, Seongnam, South Korea; 4 Department of Surgery, Seoul National University Bundang Hospital, Seoul National University College of Medicine, Seongnam, South Korea

**Keywords:** Gastric cancer, Tumour biomarkers, Tumour immunology

## Abstract

**Background:**

The aim of the study was to determine the human leucocyte antigen class-I (HLA-I), programmed death-ligand 1 (PD-L1) expression and tumour-infiltrating lymphocytes (TILs) of microsatellite instability-high gastric cancer.

**Methods:**

The HLA-I expression type was determined by immunohistochemistry of HLA-A, HLA-B, HLA-C and β2-microglobulin in the centre of the tumour (CT) and in the invasive margin (IM) of samples from 293 patients (total loss vs. preserved type). PD-L1 expression and TIL density was examined immunohistochemically. HLA-I genotyping was also performed.

**Results:**

The expression loss of the HLA-I molecules was significantly associated with low TIL density. According to survival analyses, the HLA-I expression type and PD-L1 positivity were not independent prognostic factors. The TIL density had no prognostic implication when survival analysis was performed for the whole patient group; however, high CD8^+^ TIL infiltration was significantly associated with good prognosis in only HLA-I-preserved-type/PD-L1-positive group (*p* = 0.034). The homozygosity of the HLA-I allele was more frequently observed in the total loss type group.

**Conclusions:**

We confirmed differential prognostic implication of CD8^+^ TILs according to the HLA-I and PD-L1 expression. Determination of the HLA-I expression could be helpful to select patients who would benefit from anti-PD-1/PD-L1 therapy.

## Background

Tumour-infiltrating lymphocytes (TILs) play an essential role in the defence against cancer surveillance, and their importance has been steadily raised in several malignancies.^1–3^ Within the immune system, cytotoxic CD8^+^ T lymphocytes are crucial for adaptive immunity and are activated upon recognition of peptides displayed by human leucocyte antigen class-I (HLA-I) molecules at the surfaces of antigen-presenting cells (APCs).^4^ The HLA-I molecule consists of a β2-microglobulin (B2M) light chain and a heavy chain, which is encoded by *HLA-A*, *HLA-B* and *HLA-C* genes.^5^ Cancer cells also express HLA-I molecules on their cell surface and present their tumour-specific antigens. To date, loss or down-regulation of the HLA-I molecules in tumour cells has been reported in a variety of human malignancies as one of the main mechanisms of cancer cells to escape from the anti-tumour T cell immunity.^6–8^ Alteration of HLA gene transcription, translation or post-transcriptional modification, as well as mutation of the B2M gene, might cause HLA-I down-regulation.^9,10^

Tumour-infiltrating T cells are effectors that kill cancer cells during PD-1 blockade therapy.^11,12^ Because HLA-I molecule has the crucial function of cytotoxic T lymphocytes activation, a clinical association between the HLA-I molecule and immune checkpoint blockade therapy has been suggested. A recent study reported down-regulation of HLA-I molecule in a patient’s progressed tumour lesion after the PD-1 blockade treatment, even though the baseline tumour presented diffuse strong expression of the HLA-I molecule.^13^ From these results, the authors suggested that down-regulation of the HLA-I expression could be a potential mechanism of resistance to PD-1 blockade therapy. Another study indicated that the HLA-I genotype and zygosity influenced treatment resistance to immune checkpoint blockade therapy.^14^ However, the mechanism of treatment resistance after immunotherapy and its clinical evidence have not been fully established.

Microsatellite instability-high (MSI-H) gastric cancer (GC) is associated with an increased number of mutations per tumour.^15,16^ The more mutations a tumour harbours, the more tumour-specific neoantigens are expressed, increasing the chances to be recognised by T lymphocytes.^17^ Hence, hypermutated tumours, such as MSI-H GCs, usually have high infiltration of TILs. In a recent paper Liu et al.^18^ reported that MSI-H GC is characterised by high gene expression scores for CD8^+^ T cells and interferon-γ (IFN-γ) signatures, which indicated the immunogenicity of MSI-H GC.

High prevalence of PD-L1 positivity has also been reported in MSI-H GC as well as the high infiltration of TILs.^18^ Due to these properties, MSI-H GCs are expected to respond to immune checkpoint blockade therapy, especially to the anti-PD-1/PD-L1 therapy. In the KEYNOTE-059 trial, among the GC patients who received pembrolizumab treatment, PD-L1-positive patients showed a considerably high overall response rate (ORR) (15.5%) compared to PD-L1-negative patients (6.4%).^19^ In addition, patients with MSI-H tumours experienced higher ORR than patients with non-MSI-H tumours (MSI-H vs. non-NSI-H; 57.1% vs. 9.0%). Based on this study, the US Food and Drug Administration (FDA) granted accelerated approval of pembrolizumab for treatment of GC patients, whose tumours express PD‐L1, as determined by an FDA‐approved test.^20^

The KEYNOTE-059 trial provided hoping results for GC patients; however, there are still a considerable number of non-responders in MSI-H- or PD-L1-positive groups for unknown reasons. For a more successful patient selection, the mechanism of treatment failure should be elucidated. To this end, HLA-I down-regulation has been noted as a possible mechanism for anti-PD-1 therapy resistance, according to a previous study regarding malignant melanoma.^13^ However, the relationship between expression of HLA-I and PD-L1, and tumour immunity has not been fully understood, especially in GC.

In this study, we evaluated the expression of HLA-I and PD-L1 proteins in MSI-H GCs by immunohistochemistry (IHC). First, we assessed the patient’s PD-L1 status using the PD-L1 22C3 clone antibody, which is the FDA‐approved test for pembrolizumab. The patient’s PD-L1 expression status was confirmed by combined positive score (CPS) method, which is used in the KEYNOTE-059 trial. Second, we classified the patients according to HLA-I expression type and analysed its correlation with TIL density and PD-L1 expression. Third, we examined the prognostic implication of HLA-I expression, PD-L1 expression and TIL density. Lastly, the HLA-I genotyping results were analysed to distinguish allelic distribution according to the HLA-I and PD-L1 expression.

## Methods

### Patient selection

Medical records, including medical charts and pathology reports, from January 2007 to December 2013 were retrospectively reviewed, and 3203 consecutive GC cases that had been surgically resected in Seoul National University Bundang Hospital (Seongnam-si, Republic of Korea) were collected. Among these patients, 2697 cases were microsatellite stable (MSS) disease, 190 cases were MSI-low and 316 cases were MSI-H. Out of 316 cases, 23 (7.3%) cases were excluded due to tissue unavailability. Thus, a total of 293 (92.7%) MSI-H GC cases were included in the present study. None of the patients had taken preoperative treatment. The median follow-up period was 67.1 months (range, 0.1–123.4 months). Cases lost during follow-up or due to deaths from causes other than GC were considered censored data for the survival analysis. The overall survival (OS) data were also obtained retrospectively from medical records. OS was defined as the time from surgery to the date of death. The study was approved by the Institutional Review Board of Seoul National University Bundang Hospital (reference: B-1702/383-301) and was performed in accordance with the recommendations of the Declaration of Helsinki for biomedical research involving human subjects. The Institutional Review Board waived the need of written informed consent for this study under the condition of anonymisation and no intervention to the participants.

### MSI test

All cases were tested for MSI status by the polymerase chain reaction (PCR) based on comparison with allelic profiles of five microsatellite markers (BAT-26, BAT-25, D5S346, D17S250 and S2S123) in the tumour and corresponding normal samples. The PCR products for formalin-fixed paraffin-embedded (FFPE) tissues were analysed using the ABI 3731 genetic analyser (Applied Biosystems, Foster City, CA), according to a previously described protocol.^21^

### Tissue microarray construction

Samples from the 293 surgically resected tumours were processed into FFPE blocks. Later, core tissue biopsies (2 mm in diameter) at the centre of the tumour (CT) and invasive margin (IM) were taken from individual FFPE blocks and rearranged in new tissue array blocks using a trephine apparatus (Superbiochips Laboratories, Seoul, South Korea).^22^ All immunohistochemical parameters were evaluated in the CT and IM per case.

### Immunohistochemical analysis

The expression of HLA-I complex and its subunits were confirmed by IHC using the OPTIVIEW universal DAB kit (Ventana), the Ventana Bench mark XT autostainer (Ventana) and antibodies against HLA-ABC (EMR8-5, 1:8000, Abcam, Cambridge, UK), HLA-A (EP1395Y, 1:5000, Abcam), HLA-B (1:700, Abcam), HLA-C (1:1000, Abcam) and B2M (B2M/961, 1:2000, Abcam). Immunostaining of TILs was performed with antibodies specific to CD3 (1:100; Dako, Glostrup, Denmark) and CD8 (1:100; Dako) using the Bond polymer kit (Leica Microsystems) and Leica BOND-MAX autostainer (Leica Microsystems). IHC of PD-L1 was performed on the Autostainer Link 48 with EnVision DAB Detection System (Agilent Technologies, Santa Clara, CA) and 22C3 pharmDx antibody (prediluted; Dako), according to the manufacturer’s instructions.

### Interpretation of PD-L1 expression and TIL density

**Fig. 1:**
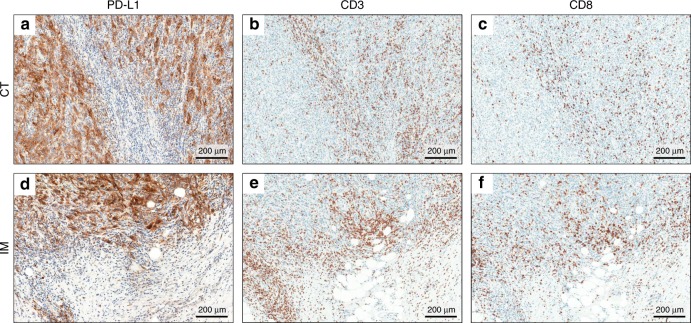
Immunohistochemistry results of a representative case. **a**, **d** PD-L1 expression by tumour and immune cells was observed both in the CT and IM of a representative case (×100). This case was PD-L1 CPS and TPS positive, both in CT and IM. **b**, **c**, **e**, **f** Immunohistochemistry confirmed the presence of CD3^+^ and CD8^+^ TILs in the CT and IM of a representative case (×100).

PD-L1 positivity was evaluated using two approaches: CPS and tumour proportion score (TPS). The CPS of the specimen was defined by the number of PD-L1-stained cells, including tumour cells, lymphocytes and macrophages, divided by the total number of viable tumour cells, multiplied by 100.^19^ PD-L1-positive tumour cells and immune cells were distinguished by their cellular morphology. The sample was considered PD-L1 positive if CPS ≥1. The TPS represented the proportion of tumour cells with moderate (2+) or higher staining intensity per total viable tumour cells. When PD-L1 TPS >1%, the sample was considered PD-L1 positive. Representative CPS- and TPS-positive IHC results are shown in Fig. 1.

For evaluation of TIL density, CD3- and CD8-immunostained slides were scanned on a slide scanner (Aperio Technologies, Aperio ScanScope^®^ Inc., Vista, CA, USA) at ×20 magnifications. CD3^+^ and CD8^+^ TIL were quantified by the computerised image analysis system, ImageScope^TM^ (Aperio Technologies) using the Nuclear v9 algorithm. The density of immune infiltrates was obtained from the entire area of the tissue core. The median TIL density was set as the cut-off value, and thereby dividing TIL density into two groups: high and low.

### HLA-I expression type and genotype analysis

**Fig. 2:**
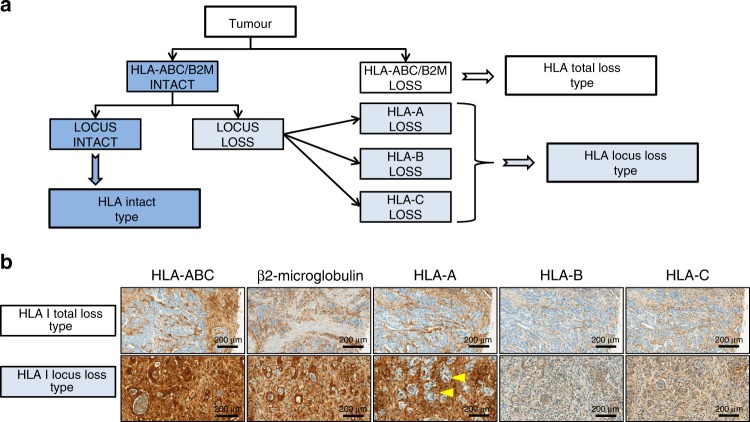
Assessment of HLA-I expression type. **a** HLA-I expression type was assessed by expression patterns of HLA-ABC, β2-microglobulin (B2M), HLA-A, HLA-B and HLA-C immunohistochemistry. First, the case showing expression loss of HLA-ABC and B2M was classified as the HLA-I total loss type. In the second stage, the HLA-ABC/B2M-positive group was sub-classified according to their reactivities to HLA-A, HLA-B and HLA-C. If a case showed expression loss of HLA-A, HLA-B or HLA-C, it was considered as the HLA-I locus loss type. No expression loss of HLA-I or its subunit was classified as HLA-I intact type. **b** Representative immunohistochemistry results of the HLA-I total and locus loss types (HLA-A locus loss) (×100). The yellow arrowhead indicates the expression loss of HLA-A in tumour cells.

All cases were classified into three HLA-I expression types according to expression patterns of HLA-I and its subunits (Fig. 2).^23^ In the first stage, patients were divided into positive or negative group based on presence or absence of HLA-ABC and B2M reactivities, respectively. In the case of HLA-ABC and B2M expression loss, the sample was classified as ‘HLA-I total loss type’. In the presence of both HLA-ABC and B2M, the sample was classified as ‘HLA-I intact type’. Samples positive for the HLA-ABC/B2M expression but with loss of HLA-A, HLA-B or HLA-C were denominated as ‘HLA-I locus loss type’.

The HLA-I genotyping was performed using 34 of 293 patients’ stored peripheral blood samples. Genomic DNA was extracted from blood using a QIAamp Blood mini kit. We genotyped HLA-A, HLA-B and HLA-C alleles based on a sequence-specific oligonucleotide amplification method using LIFECODES HLA-A, HLA-B and HLA-C eRES typing kit (Immucor, GA, USA), according to the manufacturer’s instruction. Specifically, 17 out of 34 cases were classified as HLA-I total loss type based on IHC results, and the remaining cases were HLA-I intact type.

### cBioPortal database analysis

RSEM (RNA-sequencing by expectation-maximisation)-normalised expression data (RNA Seq V2 RSEM) of 440 gastric adenocarcinoma samples were downloaded from The Cancer Genome Atlas (TCGA) via cBioPortal (http://www.cbioportal.org).^15,18^ The dataset included expression of PD-L1, CD3D, CD3E, CD8A, CD8B, HLA-A, HLA-B, HLA-C and B2M. Clinical data including MSI status and survival data on the same dataset were also obtained. Out of 440 subjects, 74 was MSI-H gastric adenocarcinoma and this dataset was selected for further analysis.

### Statistical analysis

To compare each continuous variable, a Wilcoxon’s signed-rank test was used. Correlation analysis between parametric variables was performed using Pearson’s correlation test. Meanwhile, differences between groups were compared using the log-rank test. The Kaplan–Meier method was applied to examine survival outcomes. Univariate and multivariate regression analyses were performed using Cox proportional hazards models to determine hazard ratios. *P* values of <0.05 were considered statistically significant. All statistical analyses were performed using IBM SPSS statistics 22 (Armonk, NY, USA).

## Results

### Patient characteristics and tumour immunohistochemical features

Of the 293 patients, 180 were men (61.4%) and 113 were women (38.6%). The mean age was 67.36 ± 9.69 years. Among these patients, 48.1% (141/293) and 51.9% (152/293) were diagnosed with early GC (EGC) and advanced GC (AGC), respectively. The detailed clinicopathological characteristics are presented in Table 1.

**Table 1:** Clinicopathological demographics of 293 patients.

Characteristics	*N* (%)
Age (years)	
<65	96 (32.8)
≥65	197 (67.2)
Sex	
Male	180 (61.4)
Female	113 (38.6)
pT stage	
pT1	141 (48.1)
pT2–4	152 (51.9)
pN stage	
pN0	178 (60.8)
pN1–3	115 (39.2)
Metastasis	
Absent	286 (97.6)
Present	7 (2.4)
Histologic grade	
WD	38 (13.0)
MD	151 (51.5)
PD	77 (26.3)
NA	27 (9.2)
Lauren classification	
Intestinal	226 (77.1)
Diffuse	67 (22.9)
Tumour border	
Infiltrative	118 (40.3)
Expanding	175 (59.7)
Lymphatic invasion	
Absent	128 (43.7)
Present	165 (56.3)
Vascular invasion	
Absent	273 (93.2)
Present	20 (6.8)
Perineural invasion	
Absent	239 (81.6)
Present	54 (18.4)

*WD* well differentiated, *MD* moderately differentiated, *PD* poorly differentiated, *NA* not applicable.

Among these cases, the distribution of HLA-I expression at the CT was as follows: HLA-I total loss type: 132 (45.1%), HLA-I locus loss type: 73 (24.9%), and HLA-I intact type: 88 (30.0%) cases. The HLA-I expression at the IM was as follows: HLA-I total loss: 110 (37.5%), HLA-I locus loss: 67 (22.9%) and HLA-I intact: 116 (39.6%) cases.

PD-L1 CPS positivity in the CT and IM was observed in 164 (56.0%) and 160 (54.6%) patients, respectively. Out of 293 patients, 192 (65.5%) presented PD-L1 CPS positivity in the CT or IM. Meanwhile, PD-L1 TPS positivity was observed in fewer numbers compared to CPS positivity, 96 (32.8%) and 86 (29.4%) cases in CT and IM, respectively.

CD3^+^ and CD8^+^ TIL densities were dichotomised into high- and low-density groups, according to the median values in CT and IM locations. The median values of CD3^+^ TIL densities in the CT and IM were 294.58 and 357.83 cell/mm^2^, respectively. Meanwhile, the median values of CD8^+^ TIL densities in the CT and IM were 288.68 and 385.49 cell/mm^2^, respectively. Based on CD3 IHC results in the CT, 146 (49.8%) and 147 (50.2%) patients were in TIL high- and the low-density groups, respectively. For CD8 IHC results in the CT, 147 (50.2%) and 146 (49.8%) patients were in the TIL high- and low-density groups, respectively.

### TIL densities according to HLA-I expression type and PD-L1 expression status

**Fig. 3:**
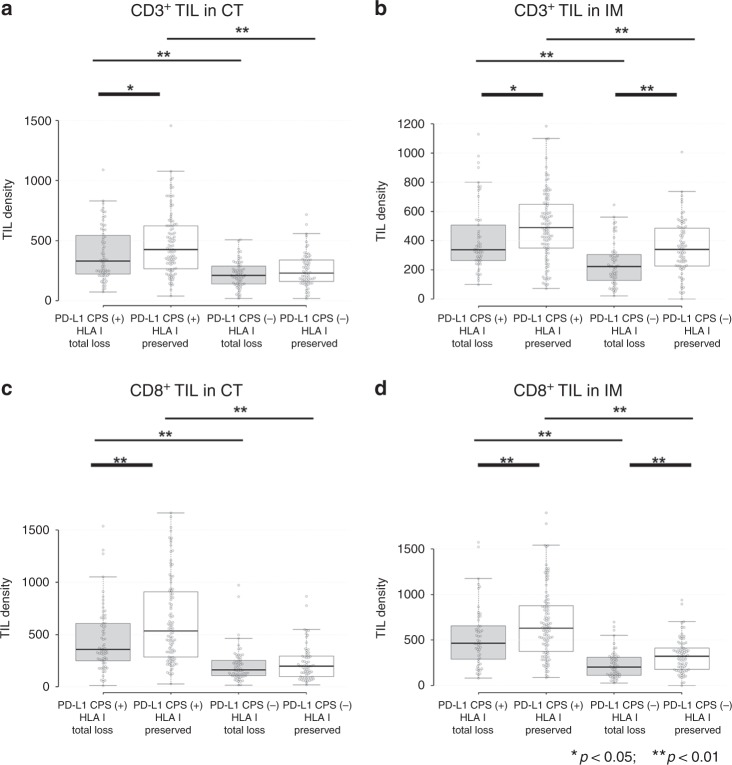
Differentiated tumour-infiltrating lymphocytes (TILs) densities according to HLA-I expression type and PD-L1 CPS status. The distribution of TIL densities according to tumour location is shown here. **a** CD3^+^ TIL in the centre of tumour (CT), **b** CD3^+^ TIL in the invasive margin, **c** CD8^+^ TIL in the CT and **d** CD8^+^ TIL in the invasive margin (IM). In the CT, the density of CD3^+^ and CD8^+^ TIL was significantly lower in HLA-I total loss type of the PD-L1 combined positive score (CPS)-positive case (**a**, **c**). In the IM, the density of CD3^+^ and CD8^+^ TIL was significantly lower in HLA-I total loss type, independently of PD-L1 CPS positivity (**b**, **d**).

In the present study, the difference of TIL density was investigated according to the HLA-I and PD-L1 expression (Fig. 3). HLA-I expression type was re-grouped into HLA-I total loss and HLA-I preserved (HLA-I locus loss + HLA-I intact). In the CT, the densities of CD3^+^ and CD8^+^ TIL were significantly lower in the HLA-I total loss type than the HLA-I preserved type in the population of PD-L1 CPS-positive group (*p* = 0.026 and *p* = 0.004, CD3^+^ and CD8^+^ TIL, respectively). However, there was no significant difference of TIL density between the HLA-I total loss and preserved type in the PD-L1 CPS-negative group in the CT. In the IM, the densities of CD3^+^ and CD8^+^ TIL were significantly lower in HLA-I total loss type than the HLA-I preserved type, in both PD-L1 CPS-positive group (*p* = 0.009 and *p* = 0.009, CD3^+^ and CD8^+^ TIL, respectively) or negative group (*p* < 0.001 and *p* = 0.001, CD3^+^ and CD8^+^ TIL, respectively). The TIL density difference according to PD-L1 TPS results showed similar tendency compared to the results obtained for PD-L1 CPS (Supplementary Fig. S1).

### HLA-I expression type and clinicopathological correlation

The detailed cross table showing clinicopathological correlation is listed in Table 2. The HLA-I total loss type was not significantly associated with aggressive clinical features, such as higher pT/N stage or distant metastasis. The proportions of HLA-I total loss type at the CT were similar, 43.3% and 46.7% in EGC and AGC patients, respectively (*p* = 0.317). Similar results were observed in the IM (HLA-I total loss type, 32.6% vs. 42.1%, EGC vs. AGC, respectively; *p* = 0.116). There was no correlation between HLA-I expression type and PD-L1 CPS positivity (*p* = 0.101 and *p* = 0.089, CT and IM, respectively). HLA-I expression type and PD-L1 TPS positivity in IM were statistically correlated (*p* = 0.002). On the other hand, no correlation was observed between HLA-I expression type and PD-L1 TPS positivity in CT (*p* = 0.117). Lower CD3^+^ and CD8^+^ TIL densities were significantly correlated with the HLA-I total loss type, regardless of tumour location.

**Table 2:** Clinicopathologic correlation of HLA-I expression type accordign to the tumour location.

	Centre of tumour				Invasive margin			
	HLA-I preserved	HLA-I total loss	Total	*P* value	HLA-I preserved	HLA-I total loss	Total	*P* value
Age								
<65	51 (53.1%)	45 (46.9%)	96	0.377	60 (62.5%)	36 (37.5%)	96	1.000
≥65	110 (55.8%)	87 (44.2%)	197		123 (62.4%)	74 (37.6%)	197	
Sex								
Male	99 (55.0%)	81 (45.0%)	180	0.539	114 (63.3%)	66 (36.7%)	180	0.711
Female	62 (54.9%)	51 (45.1%)	113		69 (61.1%)	44 (38.9%)	113	
pT stage								
pT1	80 (56.7%)	61 (43.3%)	141	0.317	95 (67.4%)	46 (32.6%)	141	0.116
pT2–4	81 (53.3%)	71 (46.7%)	152		88 (57.9%)	64 (42.1%)	152	
pN stage								
pN0	99 (55.6%)	79 (44.4%)	178	0.434	115 (64.6%)	63 (35.4%)	178	0.388
pN1–3	62 (53.9%)	53 (46.1%)	115		68 (59.1%)	47 (40.9%)	115	
Distant metastasis								
Absent	158 (44.8%)	128 (55.3%)	286	0.392	178 (62.2%)	108 (37.8%)	286	0.715
Present	4 (57.1%)	3 (42.9%)	7		5 (71.4%)	2	7	
CD3^+^ TIL density								
Low	67 (45.6%)	80 (54.4%)	147	**0.001**	71 (48.3%)	76 (51.7%)	147	**<0.001**
High	94 (64.4%)	52 (35.6%)	146		112 (76.7%)	34 (23.3%)	146	
CD8^+^ TIL density								
Low	71 (48.6%)	75 (51.4%)	146	**0.002**	80 (54.4%)	67 (45.6%)	147	**0.005**
High	90 (61.2%)	57 (38.8%)	147		103 (70.5%)	43 (29.5%)	146	
PD-L1 CPS								
Negative	65 (50.4%)	64 (49.6%)	129	0.101	77 (57.9%)	56 (42.1%)	133	0.089
Positive	96 (58.5%)	68 (41.5%)	164		106 (66.3%)	54 (33.8%)	160	
PD-L1 TPS								
Negative	103 (52.3%)	94 (47.7%)	197	0.117	118 (57.0%)	89 (43.0%)	207	**0.002**
Positive	58 (60.4%)	38 (39.6%)	96		65 (75.6%)	21 (24.4%)	86	
Total	161	132	293		183	110	293	

Bold values indicate statistical significance *P* < 0.05.

### Prognostic association of HLA-I expression type, PD-L1 expression and TIL density in MSI-GC

To investigate the prognostic relevance of HLA-I and PD-L1 expression, and TIL density, we examined the effects of these variables on 5-year OS, according to the different tumour locations (CT and IM). In the univariate survival analysis, HLA-I expression type and TIL density had no significant correlation with OS. On the other hand, PD-L1 TPS positivity in CT had a significant correlation with 5-year OS survival (*p* = 0.040). However, multivariate analysis revealed that PD-L1 TPS positivity in CT was not an independent prognostic factor (*p* = 0.622). The detailed results of univariate and multivariate analysis are presented in Supplementary Tables S1 and S2.

### Different prognostic implication of CD8^+^ TIL according to PD-L1 expression status and HLA-I expression type

**Fig. 4:**
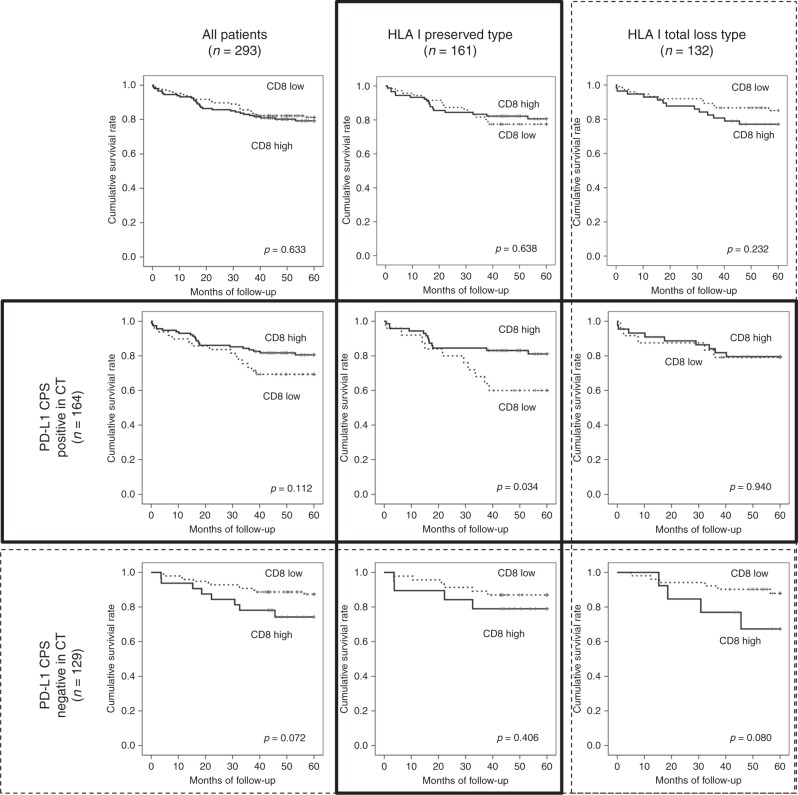
Differential prognostic implication of CD8^+^ TIL according to HLA-I expression type and PD-L1 CPS status. The prognostic association of CD8^+^ TIL was not detected in 293 patients (first row, first column). No CD8^+^ TIL prognostic impact was observed in HLA-I-preserved group or HLA-I total loss group (first row, second and third column). However, when the HLA-I-preserved group was divided into two subgroups according to PD-L1 CPS positivity, the prognostic association of CD8^+^ TIL was observed only in the HLA-I-preserved/PD-L1 CPS-positive subgroup (second row, second column). In the HLA-I total loss group, CD8^+^ TIL had no prognostic association, regardless of PD-L1 positivity (third line, left, mid and right figure).

First, we inspected the prognostic association of CD8^+^ TIL density of CT according to HLA-I expression type of CT (Fig. 4). There was no prognostic significance of CD8^+^ TIL in the HLA-I-preserved group (*n* = 161) or HLA-I total loss group (*n* = 132). Next, we subdivided the HLA-I-preserved and total loss groups into two subgroups, PD-L1 CPS positive and negative. We noticed that high CD8^+^ TIL infiltration was significantly associated with better survival in the subpopulation that presented HLA-I-preserved expression and PD-L1 CPS positive (*p* = 0.034). However, the CD8^+^ TIL infiltrates had no prognostic implication in patients who presented HLA-I total loss/PD-L1 CPS positive in the CT. This subgroup analysis was also performed for the PD-L1 TPS status. In HLA-I-preserved-type/PD-L1 TPS-positive group, high CD8^+^ TIL density was associated with a better outcome; however, there was no statistical significance (Supplementary Fig. S2).

We additionally performed multivariate analysis in the HLA-I-preserved/PD-L1 CPS-positive group and others. A prognostic significance of CD8 density was only observed in the HLA-I-preserved/PD-L1 CPS-positive group (Supplementary Table S3).

### Analysis of TCGA dataset

To validate our results that TIL densities were correlated with HLA-I expression type and PD-L1 expression status, we analysed 74 TCGA MSI-H gastric adenocarcinoma cases. In bivariate correlation analysis, the gene expression levels of CD3D and CD3E were significantly correlated with the expression of PD-L1, HLA-A, HLA-B, HLA-C and B2M expression level (Supplementary Fig. S3). CD8A expression levels had significant relation with PD-L1, HLA-C and B2M expression. CD8B expression was associated with PD-L1, HLA-A, HLA-C and B2M expression. However, PD-L1 expression levels had no correlation with any of HLA-I gene expression levels.

Next, we evaluated the prognostic significance of nine genes aforementioned TCGA dataset. Survival outcome was compared between patient group with upper quartile gene expression level and lower quartile gene expression level. According to univariate analysis, none of the nine genes had significant prognostic association in MSI-H gastric adenocarcinoma of the TCGA dataset (Supplementary Table S4).

### HLA-I genotyping results according to HLA-I expression type and PD-L1 expression

**Fig. 5:**
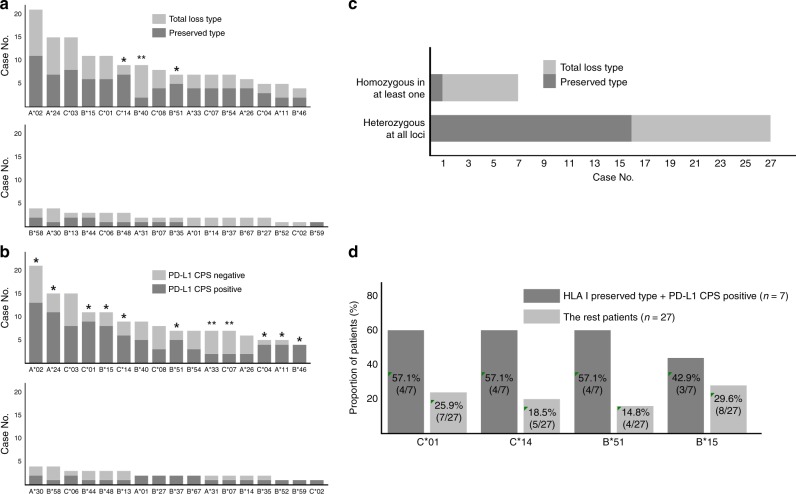
HLA-I genotyping results for 34 patients using matched blood sample. **a**, **b** The frequency of each HLA-I locus was determined according to HLA-I expression type (**a**) and PD-L1 CPS status (**b**). C*14 and B*51 were more frequently observed in HLA-I preserved type; however, B*40 was more frequently detected in the HLA-I total loss type. B*15, B*51, C*07, C*08 and C*14 were frequently observed in the PD-L1 CPS-positive group. **c** Most patients who were homozygous for at least one HLA locus were also HLA-I total loss type. **d** B*44, B*48, B*51 and C*14 were more frequently observed in the HLA-I-preserved-type/PD-L1 CPS-positive group compared to the other groups.

Out of 293 patients, we examined HLA class-I genotype of 34 patients. A simple exploration of HLA-I allele prevalence was investigated, indicating that alleles A*02, A*24 and C*03 alleles were observed at high frequencies in all examined cases. The detailed distribution of HLA-I alleles is described in Fig. 5.

When HLA-I alleles were classified according to HLA-I expression type and PD-L1 CPS positivity, some alleles showed different distributions (Fig. 5a, b). The C*14 allele was more frequently observed in patients with preserved HLA-I expression (seven patients in preserved type vs. two patients in total loss type). Similar tendency was also observed in the B*51 allele case (five patients in preserved type vs. two patients in total loss type). Conversely, the B*40 allele was presented more frequently in the total loss type (2 patients in preserved type vs. 7 patients in total loss type). The A*02, A*11, A*24, B*15, B*46 B*51, C*01, C*04 and C*14 alleles were observed in higher prevalence in the PD-L1 CPS-positive group. The A*33 and C*07 alleles were observed more frequently in the PD-L1 CPS-negative group.

Out of 34 patients, seven were HLA class-I homozygous in at least one locus (Fig. 5c). Additionally, we noticed that the total loss of HLA-I expression occurred in a higher proportion of patients with HLA-I homozygosity (six patients of total loss type vs. one patient of preserved type) compared with the remaining patients with HLA-I heterozygosity at all loci (11 patients of total loss type vs. 16 patients of preserved type). According to *χ*^2^ analysis, there was statistical significance between HLA-I expression type and HLA-I zygosity (*p* = 0.034).

Because we found a prognostic association of the CD8^+^ TIL only in the subgroup showing HLA-I-preserved and PD-L1-positive expression, we compared the HLA-I allele distribution in the HLA-I-preserved-type/PD-L1 CPS-positive subgroup (*n* = 7) and the remaining patients (*n* = 27). Of all the HLA-I alleles, four HLA-I alleles (B*15, B*51, C*01 and C*14) were frequently observed in the HLA-I-preserved-type/PD-L1 CPS-positive group (Fig. 5d). However, correlation analysis confirmed only statistical significance with B*51 and C*14 alleles (*p* = 0.039 and 0.026, respectively).

## Discussion

MSI-H GC has been steadily considered as a good responder to anti-PD-1/PD-L1 immunotherapy. Recent studies have suggested that HLA-I down-regulation can be a mechanism of treatment resistance to immune checkpoint blockade therapy.^13^ However, the clinical evidence of its role in MSI-H GCs is still lacking. There have been a few studies of HLA-I down-regulation in MSI-H colon cancers, suggesting the strong correlation between the HLA-I expression loss and B2M gene mutation, which was more frequently observed in MSI-H cancer, compared to MSS cancer.^24–26^ However, most of the researches only focused on the correlation between the HLA-I expression and other clinicopathologic factors, such as immune cell infiltration or other relevant gene mutation, without clinical outcome data.^25,26^ To the best of our knowledge, the present study is the first report that identified HLA-I expression in MSI-H GCs with its clinical implication and other cancer immune response factors, including PD-L1 and TIL infiltration. Our report provides comprehensive data encompassing PD-L1 expression and TIL densities according to HLA-I expression, which might be helpful for understanding the resistance mechanism to anti-PD-1/PD-L1 immunotherapy.

The down-regulation of the HLA-I has been reported in various malignancies, including lung, breast, oesophageal, GC, by several previous studies with diverse prevalence (27.0–65.3%).^27–32^ Furthermore, the correlation between high TIL density and preserved HLA-I expression was determined in all previous studies; however, the prognostic association of the down-regulation of the HLA-I has been controversial. In the recent study conducted by our research group, the HLA-I expression loss was observed in 65.3% of consecutive stage II–III GC cases.^32^ Unlikely to the present study, which selectively included MSI-H GC patients, the aforementioned study confirmed the significant association between the HLA-I expression loss and poor prognosis. Considering the different patient’s characteristics of two studies, the discrepancy of prognostic impact might be because of the MSI status. In our previous study, MSS and MSI-low GC cases were included, which are known to have lower immune response and worse outcome compared to MSI-H GC. In addition, we scrutinised the prognostic association of the HLA-I expression in the TCGA dataset and obtained similar results; no prognostic significance of HLA-I expression was observed in MSI-H GCs. More published evidences is needed to validate our results, but so far, the number of researches on HLA-I expression in MSI-H cancer has been limited. Therefore, large-scale studies to verify clinical significance of HLA-I in MSI-H cancer should be followed.

Meanwhile, it is noteworthy that a significant number of patients in the EGC group showed HLA-I total expression loss, not much different from the number observed in the AGC group. Our results could suggest that the alteration of HLA-I expression can be an early event of tumour progression. However, no enough data regarding the detailed profile of HLA-I expression and its in-depth clinicopathological correlation in GCs before our present study. Hence, additional clinical evidence and functional study are required to confirm the role of HLA-I expression loss in tumour progression.

Based on the established knowledge of cancer immunology, HLA-I expression loss and PD-L1 positivity represent the main mechanism of natural adaptive tumour immune evasion.^7,33^ Previous studies have identified a significant association between HLA-I expression loss and a decreased number of TILs,^34–36^ which was also confirmed in here.

In this study, we could not confirm the prognostic significance of HLA-I expression status as well as PD-L1 expression. This prognostic association was also not found in TCGA MSI-H GC dataset. Moreover, we could not find any prognostic association of CD3^+^ or CD8^+^ TIL infiltration, although TIL has been regarded as a good prognosis in GC.^37,38^ However, the significant prognostic implication of CD8^+^ TIL was only observed in PD-L1-positive/HLA-I-preserved subgroup. Our result indicates that the CD8^+^ TIL could have different roles according to the immune context of HLA-I and PD-L1 expression, in which molecules are involved in the immune evasion mechanism. Also, we suggest that the tumour immune response could not be explained by one variable and would be understood only in the relationship of multiple factors, like in the present study, TILs, PD-L1 and HLA-I.

According to recent knowledge, the PD-L1 upregulation can be explained by two mechanisms, the innate and adaptive immune response.^39^ PD-L1 upregulation by the innate immune response resulted from the dysregulation of oncogenic signals and genetic alterations in the tumour cells. In the adaptive immune response pathway, PD-L1 is upregulated by IFN-γ.^40^ When TILs encounter a tumour antigen, they secrete IFN-γ, which leads to PD-L1 upregulation on tumour cells and surrounding immune cells. As higher is the adaptive immune reaction of TIL to tumour antigen, additional upregulation of PD-L1 occurs. While PD-L1 plays an essential role in tumour immune escape, however, its upregulation could result from the active host anti-tumour immune response by TILs. Within the active anti-tumour immunity represented by PD-L1 upregulation in tumour and immune cells, the CD8^+^ T cell could still play their role when the antigen-presenting capacity is maintained by the preserved HLA-I expression. Therefore, it is reasonable that patients with high infiltration of CD8^+^ T cell had good prognosis in the PD-L1-positive/HLA-I-preserved subgroup, as observed.

Some previous studies also focused on the relationship between immune-related factors. They have suggested a new tumour classification based on tumour microenvironment immune type, which provides a better understanding of the immune microenvironment. Specifically, it is based on the PD-L1 expression and TIL density, consisting of the following four types: type I (PD-L1^+^/TIL^high^), type II (PD-L1^−^/TIL^low^), type III (PD-L1^+^/TIL^low^) and type IV (PD-L1^−^/TIL^high^).^41–43^ According to this classification, the type I tumours are the most susceptible to anti-PD-1/PD-L1 blockade, because they have sufficient pre-existing TILs that are inactivated by PD-L1 upregulation. Meanwhile, recent emerging evidence has suggested that HLA-I expression loss could result in resistance to anti-PD-1/PD-L1 blockade; nevertheless, the tumour had PD-L1 expression or high TIL infiltration.^13,44^ In our study, we confirmed the different prognostic implication of CD8^+^ TIL according to not only PD-L1 but also to HLA-I expression, indicating the important relationship between TIL infiltration, and PD-L1 and HLA-I expression. For a complete understanding of cancer immunity and immunotherapy, the association between these three factors should be studied.

In the present study, the genotyping of the HLA-I was performed in paired patient groups (HLA-I total loss vs. preserved). A previous study performed by Chowell et al.^14^ suggested that the HLA-I genotype influenced cancer response to checkpoint blockade immunotherapy. In this study, the patients with maximal heterozygosity at HLA-I loci (‘A’, ‘B’ and ‘C’) had better response to immune checkpoint blockade therapy compared to patients who were homozygous for at least one HLA locus. Due to a limited number of analysed cases (*n* = 34), we could not confirm a prognostic correlation. However, the locus homozygosity was more frequently observed in the HLA-I total loss type. Chowel et al.^14^ also confirmed that the HLA-B44 supertype was correlated with a better prognosis. In our results, only limited number of cases were tested for HLA-I genotype. Hence, our result is not enough to validate prognostic significance of each HLA-I allele. However, we detected other HLA-I alleles, which showed a higher frequency in the HLA-I-preserved/PD-L1-positive group (B*51 and C*14 alleles). Therefore, our data might have significant implications regarding the immune checkpoint blockade therapy, thus further analysis on this subject may provide fundamental data for understanding the role of HLA-I in anti-cancer immunity.

In summary, we confirmed the HLA-I expression loss in MSI-H GC. HLA-I expression itself had no prognostic association; however, we found that the CD8^+^ TILs had differential prognostic implications according to the HLA-I and PD-L1 expression in the tumour, thus affecting the cancer immunity. Therefore, the relationship of HLA-I, PD-L1 and TILs should be evaluated in order to predict the response to immune checkpoint blockade therapy. Furthermore, it might be helpful to develop a novel strategy to decide on highly advanced patient selection criteria for specific therapy in the new era of immunotherapy and precision medicine.

## Supplementary information

Supplemental material

## Data Availability

The datasets generated during and/or analysed during the current study are available from the corresponding author on reasonable request.
