# Supplementary material for: Differential prognostic impact of CD8+ T cells based on human leucocyte antigen I and PD-L1 expression in microsatellite-unstable gastric cancer
Source: Br J Cancer. 2020 Mar 17;122(9):1399–408. doi: 10.1038/s41416-020-0793-y (PMC7189244; doi:10.1038/s41416-020-0793-y)

**Supplementary Table S1**

|  | **Univariate survival analysis** | |
| --- | --- | --- |
| Variables | HR (95% CI) | P value |
| Age (**>65** vs. ≤65) | 3.877 (1.758-8.553) | **0.001** |
| pT stage (**pT3-4** vs. pT1-2) | 3.373 (1.978-5.751) | **<0.001** |
| pN stage (**pN1-3** vs. pN0) | 2.383 (1.408-4.033) | **0.001** |
| Lymphatic invasion (**Present** vs. Absent) | 2.898 (1.561-5.382) | **0.001** |
| Vascular invasion (**Present** vs. Absent) | 3.288 (1.611-6.711) | **0.001** |
| Perineural invasion (**Present** vs. Absent) | 2.536 (1.461-4.400) | **0.001** |
| PD-L1 TPS in CT (**Positive** vs. Negative) | 1.729 (1.024-2.917) | **0.040** |
| PD-L1 CPS in CT (**Positive** vs. Negative) | 1.545 (0.897-2.661) | 0.117 |
| PD-L1 TPS in IM (**Positive** vs. Negative) | 1.522 (0.889-2.608) | 0.126 |
| PD-L1 CPS in IM (**Positive** vs. Negative) | 1.296 (0.763-2.200) | 0.337 |
| CD3 density in CT (**high** vs. low) | 0.912 (0.542-1.534) | 0.728 |
| CD3 density in IM (**high** vs. low) | 1.041 (0.619-1.750) | 0.880 |
| CD8 density in CT (**high** vs. low) | 1.135 (0.675-1.909) | 0.633 |
| CD8 density in IM (**high** vs. low) | 1.056 (0.628-1.774) | 0.838 |
| HLA-I loss in CT (**Total loss** vs. Preserved) | 0.863 (0.510-1.461) | 0.583 |
| HLA-I loss in IM (**Total loss** vs. Preserved) | 0.854 (0.496-1.471) | 0.569 |

**Supplementary Table S2**

|  | **Multivariate survival analysis** | |
| --- | --- | --- |
| Variables | HR (95% CI) | P value |
| Age (**>65** vs. ≤65) | 4.678 (2.103-10.408) | **<0.001** |
| pT stage (**pT3-4** vs. pT1-2) | 1.794 (0.869-3.703) | 0.114 |
| pN stage (**pN1-3** vs. pN0) | 1.828 (0.856-3.906) | 0.119 |
| Lymphatic invasion (**Present** vs. Absent) | 1.315 (0.686-2.522) | 0.409 |
| Vascular invasion (**Present** vs. Absent) | 2.313 (1.177-4.547) | **0.015** |
| Perineural invasion (**Present** vs. Absent) | 1.202 (0.636-2.273) | 0.570 |
| PD-L1 TPS in CT (**Positive** vs. Negative) | 1.149 (0.662-1.995) | 0.622 |

**Supplementary Table S3**

|  | **Multivariate survival analysis** | | | |
| --- | --- | --- | --- | --- |
| Variables | **PD-L1 CPS + / HLA-I preserved group** | | **Other groups** | |
|  | HR (95% CI) | P value | HR (95% CI) | P value |
| Age (**>65** vs. ≤65) | 5.882 (1.289-26.834) | **0.022** | 4.527 (1.715-11.954) | **0.002** |
| pT stage (**pT3-4** vs. pT1-2) | 2.997 (0.845-10.628) | 0.089 | 1.202 (0.472-3.061) | 0.699 |
| pN stage (**pN1-3** vs. pN0) | 0.297 (0.035-2.528) | 0.267 | 3.394 (1.382-8.335) | 0.008 |
| Lymphatic invasion (**Present** vs. Absent) | 1.360 (0.441-4.195) | 0.593 | 1.492 (0.652-3.410) | 0.343 |
| Vascular invasion (**Present** vs. Absent) | 2.288 (0.912-5.739) | 0.078 | 3.048 (1.151-8.073) | 0.025 |
| Perineural invasion (**Present** vs. Absent) | 1.074 (0.446-2.585) | 0.874 | 1.075 (0.410-2.817) | 0.883 |
| CD8 density in CT (**high** vs. low) | 0.403 (0.166-0.980) | **0.045** | 1.291 (0.648-2.571) | 0.468 |

**Supplementary Table S4**

|  | **Univariate survival analysis** | |
| --- | --- | --- |
| Variables | HR (95% CI) | P value |
| PD-L1 (**High** vs. Negative) | 0.937 (0.233-3.761) | 0.926 |
| CD3D (**High** vs. Negative) | 1.043 (0.349-3.114) | 0.940 |
| CD3E (**High** vs. Negative) | 1.242 (0.405-3.810) | 0.705 |
| CD8A (**High** vs. Negative) | 0.616 (0.183-2.073) | 0.434 |
| CD8B (**High** vs. Negative) | 1.098 (0.363-3.320) | 0.869 |
| HLA-A (**High** vs. Negative) | 1.365 (0.457-4.084) | 0.577 |
| HLA-B (**High** vs. Negative) | 0.936 (0.328-2.673) | 0.902 |
| HLA-C (**High** vs. Negative) | 1.040 (0.364-2.975) | 0.942 |
| B2M (**High** vs. Negative) | 1.055 (0.352-3.160) | 0.923 |

**Supplementary Figure S1 Differentiated tumor-infiltrating lymphocytes (TILs) densities according to HLA-I expression type and PD-L1 TPS status**

The difference of TIL density according to HLA-I expression type and PD-L1 TPS status showed similar tendency with the results for PD-L1 CPS in Figure 3. In the CT, the density of CD3^+^ and CD8^+^ TIL was significantly lower in HLA-I total loss type of the PD-L1 TPS positive case **(A and C)**. In the IM, the density of CD3^+^ and CD8^+^ TIL was significantly lower in HLA‑I total loss type of the PD-L1 TPS negative case **(B and D)**.

**
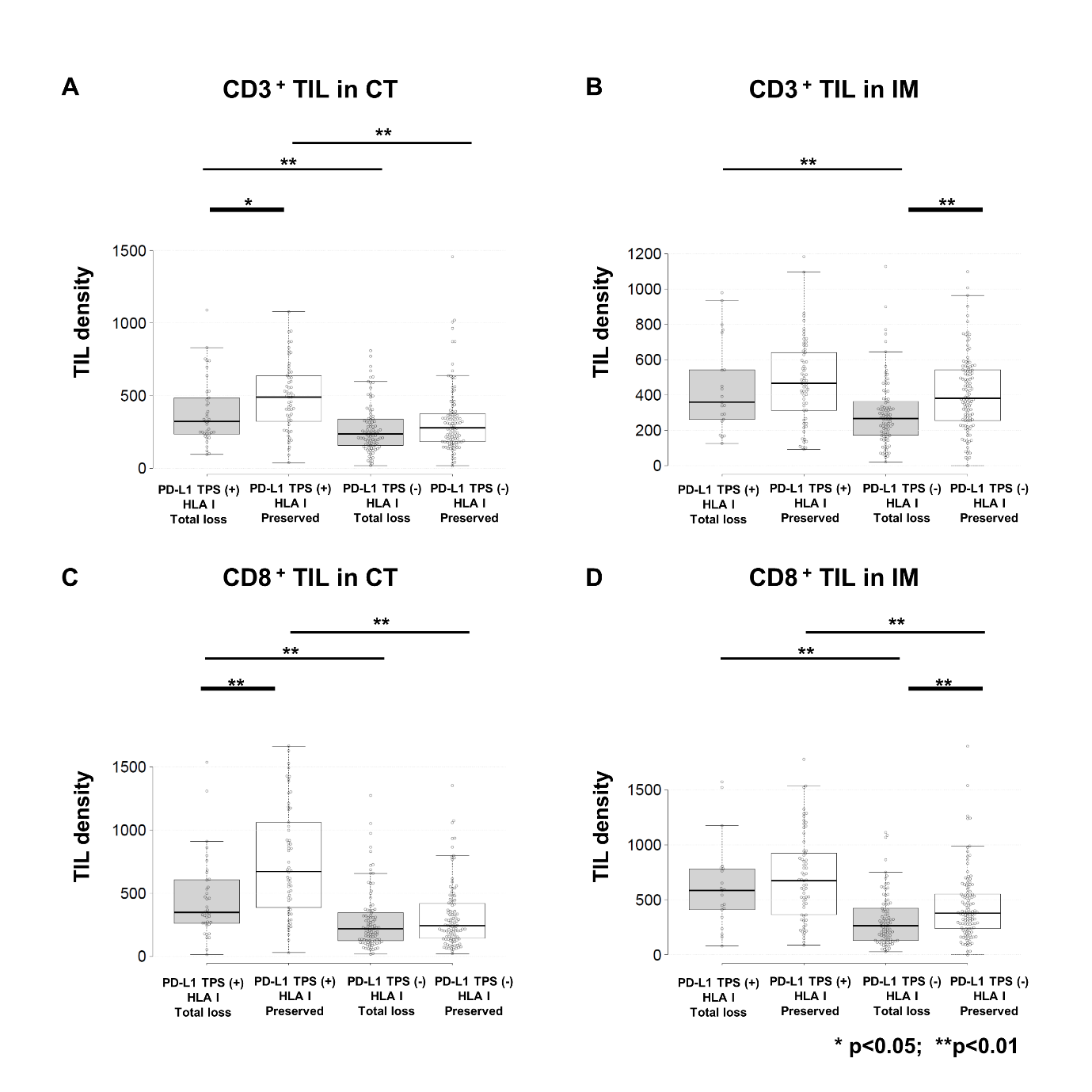
**

**Supplementary Figure S2 Differential prognostic implication of CD8+ TIL according to HLA-I expression type and PD-L1 TPS status**

The overall survival analysis showed similar tendency with the results in Figure 4. The prognostic association of CD8^+^ TIL was not found in 293 patients **(first row, first column)**. In the PD-L1 TPS positive/HLA-I preserved type group, high CD8^+^ TIL density were associated with better outcome; however, there was no statistical significance **(second row, second column)**.


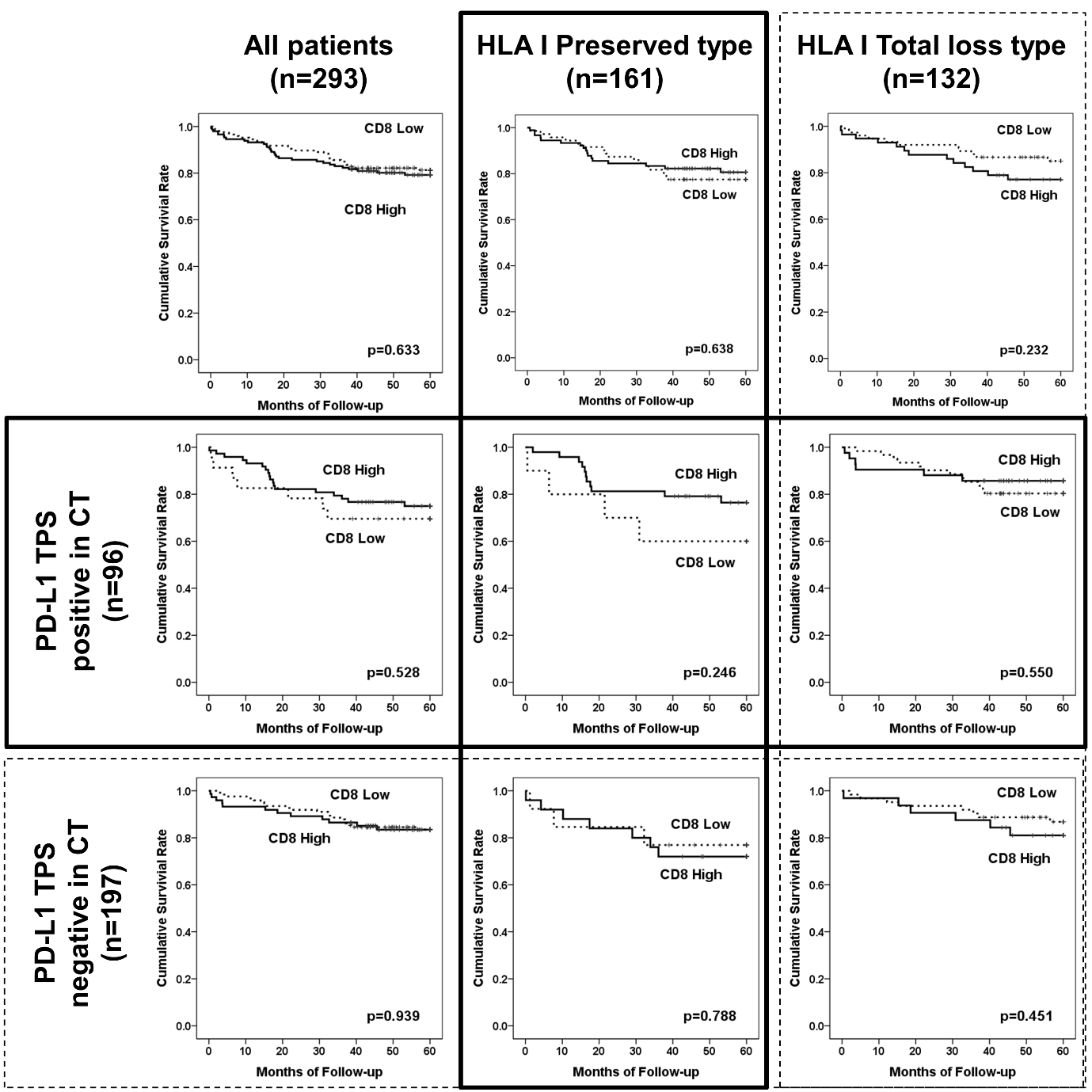


**Supplementary Figure S3**

Correlation analysis was performed between T lymphocytes (CD3D, CD3E, CD8A, and CD8B) PD-L1 and HLA-I gene sets (HLA-A, HLA-B, HLA-C, and B2M). CD3D and CD3E gene expression was significantly associated with PD-L1 and HLA-I gene set expression levels.


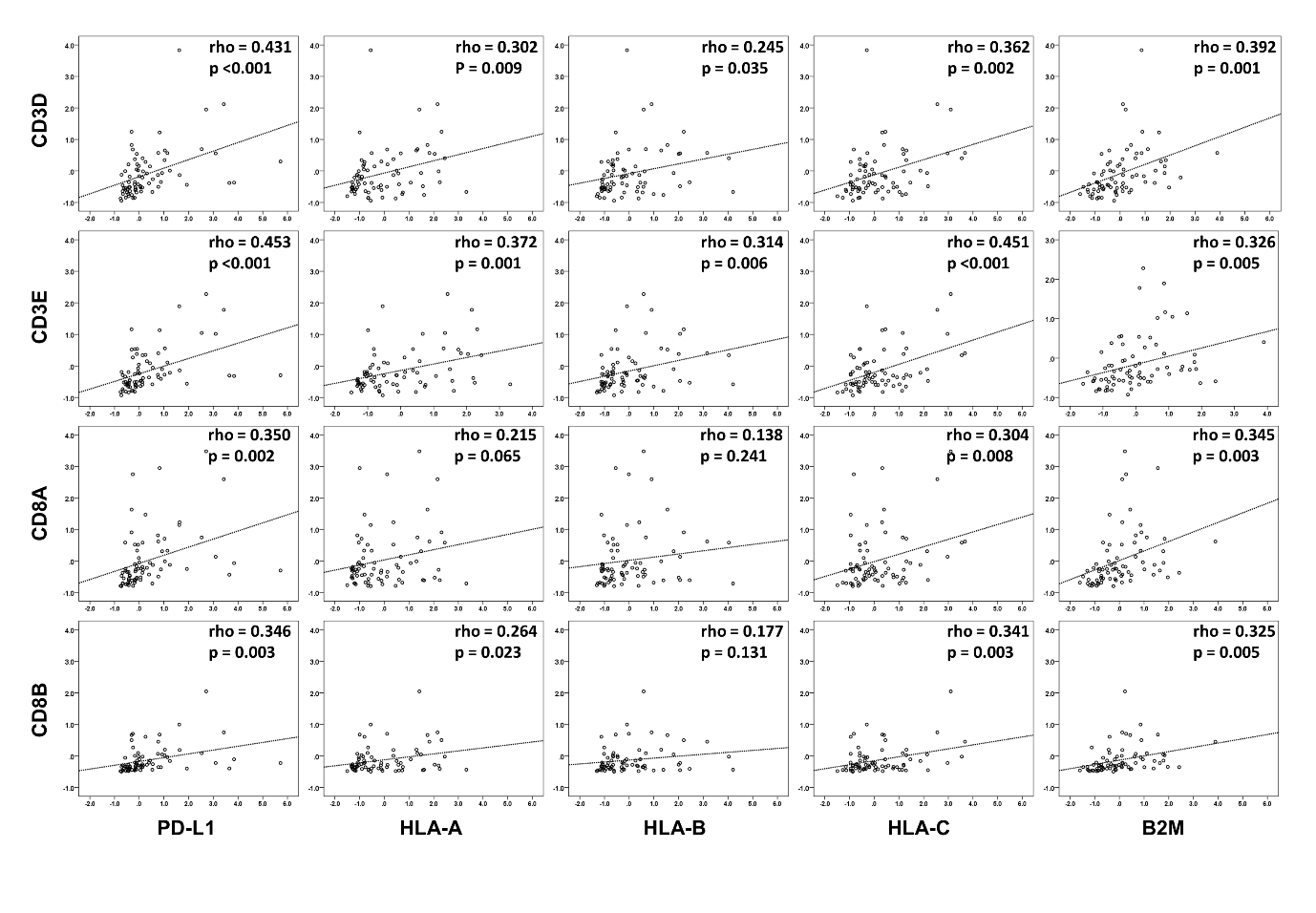

Supplement: Supplementary file 1 — Supplemental material [file 41416_2020_793_MOESM1_ESM.docx]
